# Critical Appraisal of Clinical Practice Guidelines for Age-Related Macular Degeneration

**DOI:** 10.1155/2015/710324

**Published:** 2015-05-25

**Authors:** Annie M. Wu, Connie M. Wu, Benjamin K. Young, Dominic J. Wu, Curtis E. Margo, Paul B. Greenberg

**Affiliations:** ^1^Section of Ophthalmology, Providence VA Medical Center, Providence, RI 02908, USA; ^2^Division of Ophthalmology, Warren Alpert Medical School, Brown University, Providence, RI 02903, USA; ^3^Division of Ophthalmology, Rhode Island Hospital, Providence, RI 02903, USA; ^4^Departments of Ophthalmology, Pathology and Cell Biology, Morsani College of Medicine, Tampa, FL 33612, USA

## Abstract

*Purpose*. To evaluate the methodological quality of age-related macular degeneration (AMD) clinical practice guidelines (CPGs). *Methods*. AMD CPGs published by the American Academy of Ophthalmology (AAO) and Royal College of Ophthalmologists (RCO) were appraised by independent reviewers using the Appraisal of Guidelines for Research and Evaluation (AGREE) II instrument, which comprises six domains (Scope and Purpose, Stakeholder Involvement, Rigor of Development, Clarity of Presentation, Applicability, and Editorial Independence), and an Overall Assessment score summarizing methodological quality across all domains. *Results*. Average domain scores ranged from 35% to 83% for the AAO CPG and from 17% to 83% for the RCO CPG. Intraclass correlation coefficients for the reliability of mean scores for the AAO and RCO CPGs were 0.74 and 0.88, respectively. The strongest domains were Scope and Purpose and Clarity of Presentation. The weakest were Stakeholder Involvement (AAO) and Editorial Independence (RCO). *Conclusions*. Future AMD CPGs can be improved by involving all relevant stakeholders in guideline development, ensuring transparency of guideline development and review methodology, improving guideline applicability with respect to economic considerations, and addressing potential conflict of interests within the development group.

## 1. Introduction

Blindness caused by age-related macular degeneration (AMD) affects an estimated 32.4 million people worldwide, with an additional 191 million people suffering from moderate and severe AMD-related vision impairment [1]. Therefore, it is critical to ensure that clinical practice guidelines (CPGs) for AMD abide by rigorous standards of development. However, recent studies have reported significant shortcomings in the quality of CPGs, such as a failure to clearly delineate guideline methodology, poor evidence quality, and lack of conflict of interests disclosures among guideline development group members [2–6]. In addition, there has been no recent evaluation of CPGs for AMD.

The Appraisal of Guidelines for Research and Evaluation (AGREE) II instrument is a valid and reliable tool for assessing the methodological quality of CPGs [7–9]. The original AGREE rubric along with the updated AGREE II instrument have previously been used to assess methodological quality of CPGs for the management of cataract and primary open-angle glaucoma, with shortcomings noted in a number of areas including Stakeholder Involvement, transparency of methodology, and Editorial Independence [10, 11]. We used the AGREE II instrument to evaluate AMD CPGs developed by the American Academy of Ophthalmology (AAO) and Royal College of Ophthalmologists (RCO).

## 2. Materials and Methods

The AGREE II instrument consists of 23 assessment items, with evaluators score on a scale of one (strongly disagree) to seven (strongly agree). The 23 items are organized into six quality domains: (1) Scope and Purpose; (2) Stakeholder Involvement; (3) Rigor of Development; (4) Clarity of Presentation; (5) Applicability; and (6) Editorial Independence [8]. Two final summary assessment items in the AGREE II rubric—the Overall Assessment score and reviewer recommendation of the guideline—enable reviewers to holistically evaluate each CPG.

The AAO CPG outlines recommendations for the diagnosis, treatment (nonneovascular and neovascular AMD), and follow-up care for adult AMD patients [12]. The RCO CPG outlines recommendations for AMD diagnosis, therapies for acute neovascular AMD, treatment delivery, management of nonneovascular AMD, management of chronic/long standing vision loss, referral pathways, and AMD research [13]. Four of the authors (AMW, CMW, BKY, and DJW) independently evaluated each CPG. The scores were then averaged and summarized as scaled percentage scores using the following formula given by the AGREE II: (Obtained Score − Minimum Possible Score)/(Maximum Possible Score − Minimum Possible Score) [8]. An intraclass correlation coefficient (ICC) was used to measure interrater agreement for each guideline's scores.

## 3. Results

Domain scores are summarized in Table 1; mean scores per evaluator were averaged across all items in each domain. The AAO CPG scored lowest in Domain 2 (Stakeholder Involvement) with an average score of 35% and highest in Domain 1 (Scope and Purpose) and Domain 4 (Clarity of Presentation) with scores of 83% and 79%, respectively; the average Overall Assessment was 4.5 out of 7. The RCO CPG scored lowest in Domain 6 (Editorial Independence) with 17% and highest in Domain 2 and Domain 4 with scores of 83% and 74%, respectively; the average Overall Assessment was 4 out of 7.

ICCs for the AAO and RCO CPG scores were 0.74 and 0.88, respectively. Strengths and weaknesses of the guidelines are summarized in Table 2, derived from consensus among at least three of the four reviewers in consideration of the average item scores and evaluator comments.

## 4. Discussion

To our knowledge, there have been no previous studies critically assessing multiple AMD CPGs using the AGREE II instrument. Our analysis with the AGREE II instrument outlined a number of methodological shortcomings in the guidelines.

With respect to Stakeholder Involvement (Domain 2), both CPGs failed to describe participation of patient representatives, if any, in the guideline development process. While the RCO guideline cited the involvement of one patient representative in the guideline development group, it was not possible to determine if patient groups were consulted in the development of the AAO guideline. Neither CPG detailed outcomes of public review nor how reviewer comments were used to guide the process of guideline development. An additional criterion within Domain 2 is the requirement for full disclosure of panel members' individual roles and areas of expertise. Although panel members' occupational degrees and areas of expertise were provided, CPG did not delineate panel members' specific roles within the guideline development group. Similar weaknesses in stakeholder involvement were described in the studies assessing CPGs for the management of cataract and primary open-angle glaucoma [10, 11]. Ensuring that the guideline development panel includes representatives of all stakeholders is critical in order to allow for full inclusion of relevant scientific evidence and prevent recommendation biases in favor of a particular specialty or treatment option [14]. Additionally, it has been shown that inclusion of patient views in guideline development has crucial implications for the success of guideline implementation [15, 16].

The AMD CPGs had a few shortcomings in Rigor of Development (Domain 3), the largest AGREE II domain. First, both CPGs failed to disclose details concerning external review methods, including feedback gathered from external review, patient input if any, and how the outcomes informed guideline development. Second, the CPGs lacked detailed search terms, explicit criteria for selecting evidence, names of databases searched (AAO), and dates of search (RCO). Additionally, while the AAO CPG provided an explicit rating system indicating the quality of each primary source, the RCO CPG lacked such information. Finally, the RCO CPG lacked primary source citations following each recommendation, rendering the link between evidence and recommendations unclear. Consistent citation of evidence and disclosure of evidence search methods should be incorporated into AMD CPGs to ensure rigor and transparency of evidence-based recommendation development.

Regarding Clarity of Presentation (Domain 4), recommendations were often difficult to identify within the body of each guideline due to the fact that they were either embedded within paragraphs (AAO) or listed without any direct citations of evidence or grade level indicating recommendation strength (RCO). Improving Clarity of Presentation is important as this facilitates guideline interpretation and implementation.

In Applicability (Domain 5), a common shortcoming was the lack of research on cost information and description of how economic considerations guided recommendation development. While the RCO CPG mentioned cost considerations as important factors affecting treatment adherence, few provisions were given regarding specific ways to address economic barriers to the implementation of particular recommendations in either guideline. Inclusion of cost and resource information within CPGs can facilitate transparency of treatment selection and is critically important in light of medical technology advancement amid rising healthcare costs [17]. In addition, although the AAO CPG provided summary tables, patient education materials, and a list of major recommendations to aid their implementation, the RCO CPG failed to supply such resources. The provision of such tools is critical to facilitate guideline accessibility.

Finally, the AMD CPGs had several gaps in Editorial Independence (Domain 6). Neither CPG delineated methods by which commercial interests were sought nor addressed how potential conflicts of interest (COIs) may have influenced guideline development. The AAO provides a statement on its website regarding its policy on industry relationships within clinical guideline committees [18]; however, this document does not specifically address how commercial relationships within the AAO AMD CPG development group could have influenced guideline development. Furthermore, while the AAO CPG disclosed COIs for all panel members, the RCO CPG only listed COIs of the chair. Finally, the RCO guideline did not clearly state the independence of the funding body nor describe its potential influence on guideline development. Lack of COI transparency is of particular concern when development group chairs and/or a majority of members have financial interests that are not addressed, as was the case for both AMD development groups. As potential conflicts may result in biased reporting, it is critical to ensure editorial independence and transparent COI disclosure of guideline development group members [19].

There were several potential limitations to this study. First, any rating instrument may lack objectivity. However, the AGREE II instrument has been proven to be reliable and valid, particularly with four independent reviewers. In this study, the independent reviewers were highly reliable (ICCs of 0.74 and 0.88), which is consistent with the existing research that supports the validity of AGREE II as an assessment tool [20–22]. Second, appraisals within domains such as Stakeholder Involvement, Applicability, and Editorial Independence are unable to distinguish between guidelines that gathered information and did not disclose the data from guidelines that failed to obtain any data pertaining to those domains. Although each group may have internally investigated conflict of interests and/or economic factors affecting guideline recommendations, a failure to disclose such information impairs the transparency of guideline methodology. Finally, the AGREE II assigns equal weight to each of the six domains. Since domains vary in the number of items and evaluation criteria, we used the Overall Assessment score and evaluator comments to inform comprehensive appraisals of each guideline.

## 5. Conclusions

In summary, the AGREE II evaluation of AMD CPGs highlighted methodological weaknesses in the domains of Stakeholder Involvement, Rigor of Development, Applicability, and Editorial Independence. We recommend a number of improvements for future guidelines, using whenever possible one of the two CPGs reviewed herein as a model (Table 3). Regular assessment of CPGs is important to ensure methodological rigor and quality development of guidelines offering evidence-based recommendations for clinical practice.

## Figures and Tables

**Table 1:** Comparison of AGREE II instrument evaluation scores^∗^ for age-related macular degeneration practice guidelines from the American Academy of Ophthalmology and the Royal College of Ophthalmologists.

AGREE II domain	AAO ratings					RCO ratings				
	A	B	C	D	Scaled score	A	B	C	D	Scaled score
(1) Scope and Purpose	6.0	7.0	5.3	5.7	83%	5.0	5.3	6.7	4.0	71%
(2) Stakeholder Involvement	3.7	3.7	3.0	2.0	35%	5.7	6.0	6.7	5.7	83%
(3) Rigor of Development	4.6	4.1	4.9	4.1	57%	2.9	2.6	4.4	3.0	37%
(4) Clarity of Presentation	5.7	5.7	5.7	6.0	79%	5.3	4.0	6.7	5.7	74%
(5) Applicability	5.0	3.5	3.5	2.75	45%	4.5	3.2	5.0	3.0	49%
(6) Editorial Independence	5.0	5.0	4.5	6.0	69%	1.5	1.5	2.5	2.5	17%

Overall Assessment	5	5	5	3	4.5	4	3	6	3	4

^∗^Scores were averaged across all items within each domain (maximum item score = 7).

AGREE II = Appraisal of Guidelines for Research and Evaluation II; AAO = American Academy of Ophthalmology; RCO = Royal College of Ophthalmologists.

**Table 2:** Summary of reviewers' comments organized by AGREE II domains on age-related macular degeneration clinical practice guidelines from the American Academy of Ophthalmology and the Royal College of Ophthalmologists.^*∗*^

AGREE II domain	Strengths	Weaknesses
(1) Scope and Purpose	Objectives clearly stated; specific health questions clearly described Patient population clearly specified (AAO)	None identified

(2) Stakeholder Involvement	CPG development group members' names, disciplines, institutions, and geographic locations clearly listed and easy to locate (RCO) Guideline development group included individuals from relevant professional groups, including a patient representative (RCO)	No statement of type of strategy used to capture views and preferences of the patients/public Lack of outcomes gathered from patients/public and how feedback was used in guideline development process

(3) Rigor of Development	Recommendations describing health benefits, risks, and side effects of recommendations Recommendations preceded by a section detailing pertinent evidence A primary source (AAO) cited by each recommendation Guideline externally reviewed prior to publication; next review date provided	Lack of search terms for evidence, full search strategy, time periods searched, name of databases, and/or inclusion/exclusion criteria Lack of rating system for quality of evidence; recommendations lacking direct citation of evidence (RCO) No clear description of recommendation formulation process (e.g., voting procedures) Methods/outcomes of external review not explicit

(4) Clarity of Presentation	Specific and unambiguous wording of recommendations Different options for treatment clearly presented in tables (AAO)	Key recommendations difficult to identify, particularly when embedded in main body of text or given without direct evidence citation

(5) Applicability	Tools and advice provided for recommendation implementation: list of major recommendations, treatment trial summaries, glossary, and links to patient education materials (AAO) Auditing criteria (e.g., recommended medication dosages and follow-up intervals) listed in tables (AAO)	Lack of descriptions of facilitators and barriers to application (AAO) Lack of a clear summary document of key recommendations (RCO) Resource implications not addressed: no mention of health economist as part of guideline development group, no information on types of cost data considered, and/or methods by which data was sought

(6) Editorial Independence	Independence of funding body clearly stated (AAO) Conflicts of Interest (COIs) disclosed for all group members (AAO)	Independence of funding body not clearly stated (RCO) COIs for group members other than the chair not listed (RCO) Lack of methods by which potential COIs were sought and description of how competing interests influenced the guideline development process

^*∗*^Comments specific to one CPG are indicated by parentheses. Comments lacking denotations pertain to both CPGs.

AGREE II = Appraisal of Guidelines for Research and Evaluation II; CPG = clinical practice guideline; AAO = American Academy of Ophthalmology; RCO = Royal College of Ophthalmologists.

**Table 3:** Recommendations for improvement from an AGREE II assessment of age-related macular degeneration clinical practice guidelines from the American Academy of Ophthalmology and the Royal College of Ophthalmologists.

Primary recommendations	Model from evaluated CPGs
Stakeholder involvement: clarify roles and input of patient representatives in guideline development and review	None
Transparency of guideline development process: clearly delineate roles of each member, evidence search process, methods of guideline development, and review	None
Applicability: address facilitators and barriers to application of recommendations by detailing economic factors affecting guideline implementation	None
Editorial independence: disclose methods by which commercial interests were sought and how competing interests may have influenced the recommendation formulation process	None

Additional recommendations	

Strengthen stakeholder involvement by including a patient representative and disclosing members' institutional affiliations	RCO
Improve transparency of development by including evidence ratings and direct evidence citations for each recommendation	AAO
Provide summary tables, patient education materials, and list of major recommendations to facilitate their implementation	AAO
Ensure and disclose independence of the guideline development group from potential influences of the funding body	AAO

AGREE II = Appraisal of Guidelines for Research and Evaluation II; AAO = American Academy of Ophthalmology; RCO = Royal College of Ophthalmologists.

## Presentation

Poster presentation at ARVO 2015.
